# The downward referral experiences of stroke caregivers in the regional medical alliance in China: A phenomenological study

**DOI:** 10.1097/MD.0000000000031151

**Published:** 2022-10-21

**Authors:** Yuan Wang, Lijun Xiang, Jian Chen, Yanli Cui, Fengwen Wang, Xiaomei Zhang

**Affiliations:** a Nanfang Hospital, Southern Medical University, Guangzhou, Guangdong, China; b School of Nursing, Southern Medical University, Guangzhou, Guangdong, China; c Baiyun Branch, Nanfang Hospital, Southern Medical University, Guangzhou, Guangdong, China.

**Keywords:** downward referral, experience, medical alliance, phenomenological study, stroke caregivers

## Abstract

The downward referral platform in the regional medical alliance has provided more possibilities to follow-up rehabilitation and transitional care for increasing stroke survivors, which also has the most contributions in the rational use of resources and health promotion of stroke survivors. However the downward referral rate is low compared to upward referral. At present, no scholars have explored the downward referral experiences of medical demanders from the perspective of qualitative study, and these experiences may also most truly reflect the influencing factors of their unwillingness to downward referral. Therefore, this study explored the subjective experiences of stroke caregivers who had experienced the downward referral, because stroke attacks often lead to lack of autonomy of patients themselves, making it difficult to complete interviews with them. A descriptive phenomenological study was adopted. A purposive sampling strategy was used to recruit 13 stroke caregivers. Interviews were guided by a semi-structured interview-guide encouraging interviewees to reflect on their experiences with downward referral. Coliazzi’s data analysis process was applied. The analysis of the data revealed 4 themes: coping challenges; disrupted information; gaps in medical and nursing transition, and potential enabling factors. The results of this study showed that the lack of knowledge of medical alliance, non-sharing of medical information and non-homogeneousness of medical quality were identified to be impeding positive attitude towards downward referral and be factors of bad experiences. Of course, the interviewees had positive experiences such as smooth referral and comfortable environment. These may be potential enabling factors to their attitude towards downward referral. The challenges and needs of medical demanders after downward referral are worthy of attention, and these should be solved by corresponding measures to improve the downward referral rate and referral experiences.

## 1. Introduction

The data on the global burden of stroke show that increasing attack rates and decreasing mortality have led to a rise in the number of stroke survivors.^[1]^ It is estimated that stroke incidence and survival rates will rise to 23 million and 77 million, respectively, by 2030.^[2]^ Due to Chinese limited health resources, most stroke survivors are discharged home before fully functional recovery. For example, a study^[3]^ of 9361 patients with a first stroke showed that 53.1% had functional dependence at discharge. Because these dysfunctions require long-term rehabilitation and care.^[4]^ The demand for follow-up rehabilitation and transitional care has become more prominent in the context of shortened hospital stays and early discharge. Transitional care and postdischarge care services are available in many developed nation. However, in China, some scholars found that the establishment of transitional care model with Chinese characteristics was a problem to be solved.^[5,6]^

In recent years, as the reform of public hospitals progresses, the hierarchical medical system has provided more possibilities for Chinese-style transitional care model. The medical alliance is an important carrier of the hierarchical medical system, and its realization depends on the 2-way referral system in the medical alliance. The hierarchical medical system is an essential system in many developed countries.^[7]^ For example, the United Kingdom is the first developed country in the world to implement the hierarchical medical system. Similarly, in China, hospitals are divided into 3 levels according to their functions and tasks,^[8]^ including primary hospitals, secondary hospitals and tertiary hospitals. In a region, through government policy guidance and the leading role of higher-level hospitals, these different levels of hospitals have formed a regional medical alliance with resource sharing and division of work. Two-way referral system means upward referral and downward referral. The former refers to the referral to a higher-level hospital as needed, and the latter refers to the referral to lower-level hospitals or primary care medical institutions for rehabilitation and transitional care. The downward referral provides a convenient and preexisting platform for stroke survivors in need of follow-up rehabilitation and transitional care, by the way, addressing the lack of postdischarge care services. In the past 2 years, some scholars^[9–11]^ had carried out relevant researches, and the results showed that transitional care provided via the downward referral platform could improve quality of life and disease status of stroke patients.

However, the downward referral rate is significantly lower than the upward referral rate, showing a 1-way referral situation in China.^[12]^ For example, a study conducted in 10 provinces of China in 2014 showed that the number of upward referral was 5 times that of downward referral.^[13]^ Another survey found that the proportion of patients who should be referred down was about 8.3%, while the actual ratio was only 4.25%.^[14]^ A small number of quantitative studies had explored the reasons.^[15–17]^ But the objects selected were mostly residents or patients who had not yet experienced downward referral. Little is known about the experiences from the perspective of the object who had experienced downward referral.

In addition, because stroke attacks often lead to lack of autonomy of patients themselves. And those who need a downward referral rather than a direct discharge home are usually more seriously ill, making it difficult to complete interviews with them.

Based on the above background, the stroke caregivers who had experienced the downward referral were recruited. This study would further explore the experiences of caregivers and identify the barriers and enablers relative to the downward referral. And, the data would be the empirical basis for building transitional care programs across medical institutions at different levels later. In addition, it was more important to put forward scientific suggestions for improving the implementation effect of hierarchical medical system in China. And the ultimate goal was to facilitate the system of maturity of the downward referral, thus providing a high-quality platform to patients, and enhancing their willingness and experiences with downward referral. Even, the results of this study in China could provide a reference for such developing countries.

## 2. Methods

### 2.1. Design

The descriptive phenomenological approach was applied because the aim was to describe human experiences as lived by the caregivers who experienced downward referral. And little is known about caregivers’ experiences of downward referral.

### 2.2. Participants and setting

This study was conducted in the Department of Neurology in a lower-level hospital (hospital A) from April to July 2021. With the help of the district government, this hospital established a close regional medical alliance with hospital B. The referral from hospital B to hospital A is the downward referral mentioned earlier. The purposive sampling method was used to recruit the stroke caregivers. Inclusion criteria are: informal caregivers (such as family members or relatives) and they experienced the referral from hospital B to hospital A; the caregivers participated in the whole-course nursing at 2 hospitals; and provided consent for study participation. Those paid for working with patients were excluded. The sample size was based on information saturation.^[18]^

### 2.3. Data collection

Before the interviews, the purpose and significance of this study was explained to interviewees, and they signed informed consent forms. And, their demographic data – age, sex, relationship with patient, education level, referral days, and whole-course nursing – were collected. Also, they were told that they could choose to withdraw from the study at any time and for any reason. Fortunately, none of the participants had these situations. Then, the first author conducted all face-to-face interviews. Only the interviewee and the first author attended the interview. In order to make the interview more successful, the first author had been trained in qualitative interview methods in advance. During the interview, the first author paied attention to the interviewee’s non-verbal expressions such as facial expressions, tone of voice, intonation, actions and so on, and also, wrote down these key informations in a notebook. Two pilot interviewees were conducted to enhance the study’s acceptability and credibility. These 2 interviews were not included in the final data. Table 1 shows the specific interview questions, and the content was revised by experts in psychology, qualitative specialist and the results of the pilot interviewees. All interviews were audio-recorded and lasted around 30 to 60 minutes.

**Table 1:** The Interview topic guide.

(1) How much do you know about the medical alliance and the referral system?
(2) Who recommended the downward referral to you? And, why did you accept it?
(3) How did you feel when you learned that your family members could be referred down?
(4) Did you encounter any difficulties during the referral process? If so, what are the specific difficulties? To solve these difficulties, who helped you?
(5) What do you think of the medical treatment and care your family members receive now?
(6) What other medical treatment, rehabilitation and nursing needs of you and your patient have not been met here?

### 2.4. Data analysis

**Figure 1.**
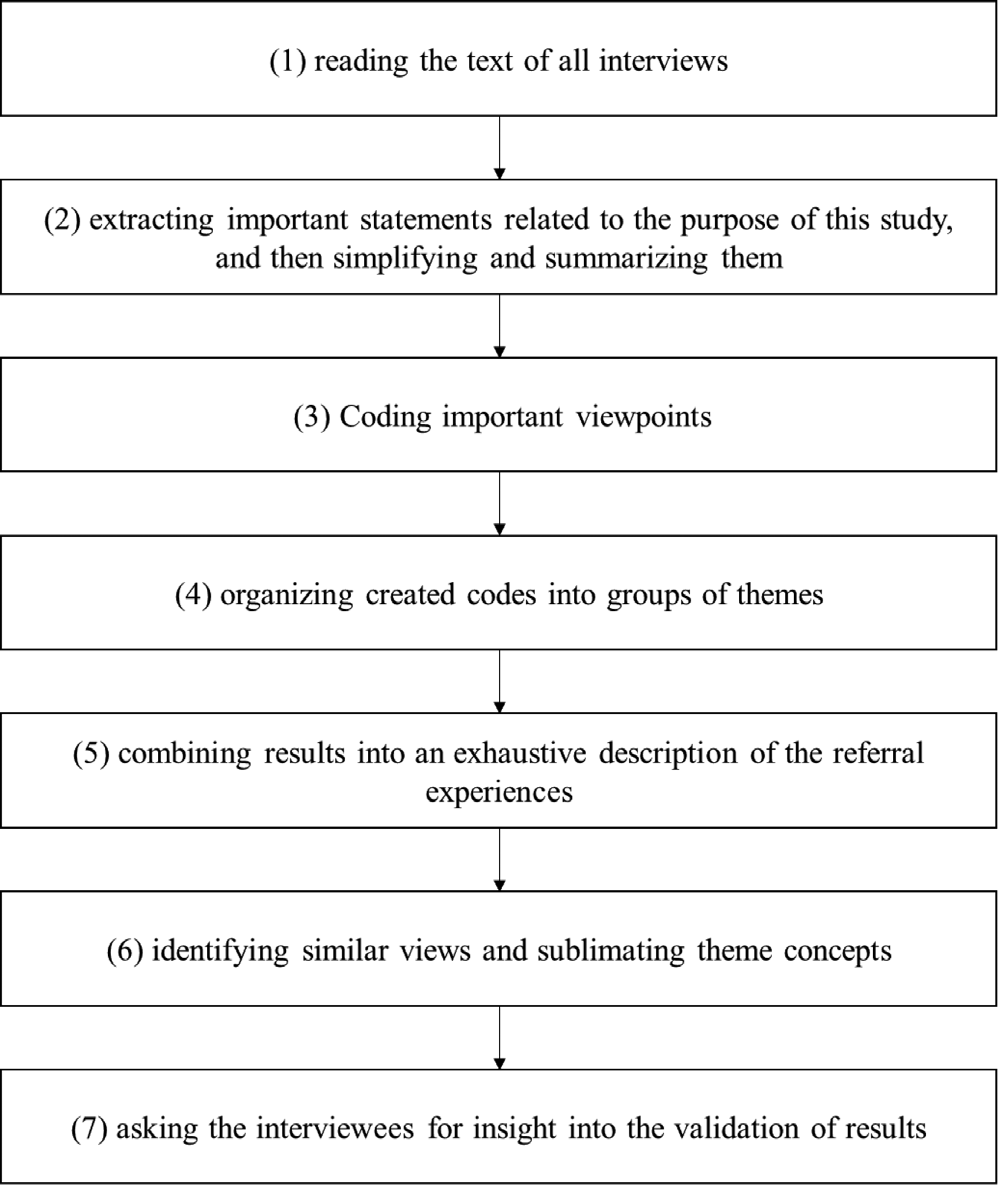
Colaizzi’s 7-step method, which is the statistical method of this study.

The interviews were audio-recorded. Within 24 hours of each interview, the audio-recorded were transcribed verbatim and collated in conjunction with on-site notes. The contents were analyzed using Colaizzi’s 7-step method,^[19]^ and the specific steps are shown in Figure 1.

### 2.5. Ethical issues

This study received approval from the Local Ethics Committee (The approval number is NFEC-2021-361). Prior to the interviews, the interviewees received written and oral information emphasizing voluntary participation and the option to withdraw at any time. Written information consent was obtained before the interviews. The researchers promised not to use any personal identifier in research reports or publications. All the interviewees were encrypted and only available to the study team.

### 2.6. Trustworthiness

The rigour of this study was enhanced in these following techniques:

1. The first author worked in the higher-level hospital in advance to establish trust relationship with patients and their caregivers.
2. During the interview, the first author maintained an objective and neutral attitude and avoided suggestive questions; in the process of data analysis, respected the original statements of the interviewees and did not mix with personal understanding and opinions; after the data analysis, the text data were handed over to the interviewees for verification to ensure the stability of the results.
3. The same interviewer (the first author) conducted all the interviews primarily, ensuring the authenticity and data saturation through prolonged engagement.

## 3. Results

### 3.1. Participants’ characteristics

A total of 13 stroke caregivers was recruited. Their ages ranged from 22 to 57 years. The detail demographic characteristics are shown in Table 2. From the analysis, 4 themes and 10 subthemes emerged, as presented in Table 3.

**Table 2:** Demographics characteristics (N = 13).

Code	Sex	Relationship with patient	Referral days	Age (yr)	Education levels	Whole-course nursing
A	Female	Adult children	7	22	Bachelor’s degree	Yes
B	Female	Adult children	5	26	Junior Secondary Schools	Yes
C	Female	Sibling	7	45	Bachelor’s degree	Yes
D	Female	Parents	6	57	Junior Secondary Schools	Yes
E	Male	Sibling	8	42	Primary Schools	Yes
F	Female	Adult children	5	32	Junior Secondary Schools	Yes
G	Male	Spouse	6	35	Senior Secondary Schools	Yes
H	Female	Sibling	5	40	Senior Secondary Schools	Yes
I	Male	Adult children	5	45	Primary Schools	Yes
J	Male	Spouse	7	48	Primary Schools	Yes
K	Female	Spouse	7	55	Primary Schools	Yes
L	Female	Adult children	5	45	junior college	Yes
M	Male	Adult children	5	36	Bachelor’s degree	Yes

**Table 3:** Themes and subthemes.

Themes	Subthemes
Coping challenges	Poor cognition
	Low subjective initiative
	Passive obedience
Disrupted information	Incomplete handover of medical information
	Seeking information
Gaps in medical and nursing transition	Dissatisfaction about less contact with doctors
	Concerns regarding medical and nursing
Potential enabling factors	Smooth referral
	Comfortable environment
	Better expectation

### 3.2. Coping challenges

#### 3.2.1. Poor cognition.

Due to the lack of publicity of medical alliance and referral system, the interviewees had poor knowledge of them. When asked whether they knew the medical alliance or referral system within it, almost all interviewees do not know, and a few interviewees superficially understood the referral system as a transfer between hospitals.

“I have not heard of them (medical alliance and referral system), and all I know about is transferring to this hospital (hospital A), just transferring.” (B)

“I have not heard of referral.” (D)

“I do not know the referral system.” (E)

“I do not understand the referral system. I have not heard of it.” (G)

“I do not know them (medical alliance and referral system).” (A, C, H-K)

“I know a thing or two about it. I do not know too much, and does it mean transfer?” (F)

“I only know the hospital transfer, and is it the referral you said?” (L)

“I only know that the doctor of hospital B let us transfer, and do not know about other things.” (M)

#### 3.2.2. Low subjective initiative.

Due to the lack of knowledge, the interviewees had low subjective initiative (attitude). When asked about feelings of knowing their family could be referred down, most interviewees were calm and a few were negative or positive.

“The doctor of hospital B said we need to come here (hospital A) to receive rehabilitation, and he can help us, so I naturally have no other ideas.” (D)

“We just followed the doctor’s advice. The doctor said that the ward environment here (hospital A) is quieter, which is conducive to her (patient) recovery, and I have no other special feelings.” (G)

“The doctor of hospital B helped me contact the hospital A, so I think it is convenient, and I have no other feelings.” (H)

“Feeling as if nothing, and we just followed the doctor”s arrangement.’ (I)

“We do what the doctor suggests.” (J)

“Feeling as if nothing.” (K, L)

An interviewee was fully psychologically prepared in advance, so she actively accepted it.

“He (patient) is on the mend. He has been laid up for 3 months, so I think it is acceptable.” (C)

An interviewee had a negative attitude towards the referral because he thought that it was troublesome.

“No matter what, it is more convenient to be in the same hospital throughout the treatment period, and it is troublesome for the downward referral.” (E)

Differently, an interviewee was happy about it.

“The downward referral proves that he (patient) is making steps in a positive direction. So when I heard it, I was actually happy.” (F)

#### 3.2.3. Passive obedience.

Because they did not know the medical alliance relationship between hospitals, no interviewees actively proposed downward referral, but passively followed the doctor’s advice unconditionally or on favorable terms.

“The downward referral was recommended by the doctor.” (C-F, H-L)

“The professor of hospital B said that this (hospital A) is a branch hospital. And he also said it was very convenient for doctors between two hospitals to talk about his (patient) condition. In my opinion, this point was very important, because I was afraid I could not completely transfer medical information to other hospitals due to the lack of medical knowledge.” (G)

“I was reluctant at first, but the doctor told us that some of the professors and doctors in hospital A were dispatched by the hospital B, which ensured the convenience of communication between doctors and the homogeneity of medical quality, so I was willing to accept it.” (M)

“If it was helpful to him (patient), I would definitely accept it.” (B)

### 3.3. Disrupted information

#### 3.3.1. Incomplete handover of medical information.

Due to the imperfect information platform in the medical alliance, medical information sharing was not a reality. There were some complaints from interviewees when medical information handover was incomplete.

“The doctor in hospital A had a consultation again, so I think that there must be a problem with the communication between doctors in the two hospitals.” (G)

“The doctors in the two hospitals should check the patient’s information by themselves and stop asking me.” (H)

“The doctors in hospital B should communicate well with the doctors here (hospital A) so that I can feel more relaxed. It is better not to ask me.” (A)

#### 3.3.2. Seeking information.

Because the information platform was not uniform, the interviewees could not obtain information in hospital A as easily as in hospital B.

“In hospital B, I could obtain information about various inspection reports, medications, and treatments performed by the doctors for my brother (patient) on the mobile phone public account every day. But, in hospital A, I cannot find a doctor to answer my questions.” (C)

### 3.4. Gaps in medical and nursing transition

#### 3.4.1. Dissatisfaction about less regular contact with doctors.

On the 1 hand, hospital B is a large-scale comprehensive tertiary first-class hospital with a large number of doctors with superb medical skills. But it is not so good in hospital A, as a result, many interviewees expressed disappointment when they were usually unable to see their doctor after downward referral.

“There are too few doctors (walking into the ward) in hospital A, and they are not as good as the doctors in hospital B.” (A)

“It’s difficult to find a doctor in hospital A. We hope that the doctor can come to the ward to see how the condition is progressing.” (I)

“I cannot find my doctor. The level of doctors in hospital B is very detailed. Every doctor knows the condition very well. This is not the case here.” (C)

“I see, most doctors in hospital A are young which makes me feel insecure.” (H)

On the other hand, the doctors in hospital B told the interviewees that they were going to make ward rounds in hospital A. Some interviewees expressed disappointment when this expectation was not met.

“The doctors from hospital B said that they would come here (hospital A) for rounds, but I have not seen them until now.” (G)

“The doctors in hospital B should take care of us every few days and dynamically observe the patient’s recovery, instead of leaving us alone.” (H)

#### 3.4.2. Concerns regarding medical and nursing.

The quality of doctors and nurses in hospital A was relatively low. Therefore, the interviewees expressed some concerns.

“I feel that there is no active treatment in hospital A, it is the same every day.” (H)

“Medical treatment is really..., (laugh helplessly) it does not seem to have any effect, and it is not very good anyway.” (F)

“Some nurses lack professional knowledge. The nurses told me very little, and only when I consulted, did she tell me something.” (A)

“The young nurses have some irregular operations and lack of asepsis awareness.” (D)

### 3.5. Potential enabling factors

In addition to the pretty terrible experiences above, there were also some good feelings. It was worth noting that what the caregivers praise was their inner needs, let alone their direct expectations, which may be potential enabling factors to their attitude towards downward referral.

#### 3.5.1. Smooth referral.

When asked if there were any difficulties in the referral process, all the interviewees said that the referral process went smoothly without any trouble.

“There was no difficulties. The ambulance took us here (hospital A) directly, and then the doctor came as soon as we arrived.” (A-M)

#### 3.5.2. Comfortable environment.

Compared with hospital B, hospital A has relatively less flow of patients and others, so the hospitalization comfort was improved.

“The environment here (hospital A) is good, and hospital B is crowded.” (E)

#### 3.5.3. Better expectation.

In the final discussion, some interviewees also expressed some expectations in order to get better experiences.

“Hospital A should expand the rehabilitation team so that we have a good experience here. The rehabilitation team is in hospital A, and the treatment team is in hospital B, which is beneficial for us.” (H)

“Nursing techniques need to be improved, such as infusion operation. And some nurses” attitude also needs to be improved.’ (G)

## 4. Discussion

### 4.1. Interpretation within the context of the wider literature

This study revealed that most caregivers had a low level of knowledge, attitude and practice related to the medical alliance and referral system, which was consistent with another study.^[20]^ As medical demander, the patients’ knowledge, attitudes and supportive behavior of the medical alliance precisely determined the degree of participation and even affected the success of the medical alliance.^[21]^ For example, according to a survey, 53.6% of patients refused downward referral because they did not sufficiently understand it.^[22]^ Differently, we took caregivers as interviewees, and the medical decisions are often made by them. Therefore, the caregivers’ knowledge, attitude and practice are equally important. The change of group behavior would go through a series of links from cognition, belief, intention to action.^[23]^ So, it’s suggested to fully explain the hierarchical medical system to patients and their caregivers to improve their awareness and acceptance, so as to change their medical notion and even medical behavior.

It is important to note that some caregivers were willing to accept the downward referral only when they knew that it enabled patients to enjoy the same level of medical services and facilitated information exchange between doctors in 2 hospitals. Actually, its role is not so simple. Firstly, from the treatment cycle, the follow-up rehabilitation and transitional care are not much profit in tertiary hospitals.^[24]^ Thus, patients during the rehabilitation period have to discharge to improve the efficiency and benefit of beds. However, a survey showed that more than 60% of stroke patients and their families were discharged without meeting their continuous care needs in terms of rehabilitation equipment instruction and home care skills.^[25]^ So, the downward referral platform providing high-quality follow-up rehabilitation and transitional care is important for stroke survivors. Secondly, individually, it plays an important role in improving clinical outcomes. Finally, at the national level, the positive attitude towards downward referral undoubtedly promotes sustainable development of medical policy of medical alliance, and at the same time, medical alliance is an important carrier and channel for implementing hierarchical medical system. Therefore, it also indirectly contributes to the implementation of medical policy of hierarchical medical system, especially helps solve the low rate of downward referral. Further, The implementation of these health policies will produce a series of favorable outcomes: promoting the rational allocation of health resources, improving the effective utilization of existing health resources, promoting the rational diversion of patients, reducing the blindness of patients’ medical treatment, balancing the supply and demand of health services, and forming a pattern of “Minor illness in the community, serious illness in hospital”. As the leading units, the large-scale comprehensive tertiary first-class hospitals monopolize a large number of high-quality medical resources, and their active participation had an important impact on the operation of the medical alliance.^[26]^ A study suggesting that patients’ attitude towards downward referral was influenced by patient–doctor communication.^[27]^ And, due to the asymmetric information between patients and doctors, patients’ choice of referral depends largely on doctors’ advice, which was also confirmed in this study.

So, it’s suggested to further strengthen the studying of medical staff on the referral system, encourage them to actively introduce its advantages to patients and families in order to increase the downward referral rate. In addition, strengthening the publicity of the medical system from the needs of the disease itself so as to increase patients and caregivers’ perception of its advantages. Finally, the government plays an important guiding role in the publicity of medical alliance. The power of policy enforcement determines the public awareness level. It is time to seriously implement government policy to increase the publicity of the medical system.

One scholar suggested that the development of medical alliance should be combined with information technology.^[28]^ The core and starting point of the medical alliance construction is resource sharing, and the informatization is the best way to promote the integration of medical resources and information sharing.^[29]^ Therefore, further implementation and improvement of relevant policies on the information platform construction would be of great significance to the promotion of hierarchical medical system.^[30]^

In this study, the initial intention of some caregivers agreeing downward referral was to worry that they couldn’t completely transfer medical information to other hospitals due to the lack of medical knowledge. If the information sharing platform is not perfect, the advantages of medical alliance cannot be effectively exerted, which would bring bad experiences to patients and their caregivers, just like the interviewees in this study. A study also showed that the timeliness and integrity of information delivery were the primary factors influencing patients’ satisfaction with the referral process.^[31]^ Without effective and efficient information sharing, medical referral will be only a bureaucratic procedure between hospitals, which is contrary to the goals and principles of medical alliance.

However, the information platform construction is not accomplished overnight. It is necessary to rely on the leadership of the government to form a government-led and multi-funded situation to establish an information platform and realize the interconnection and intercommunication of medical information in the medical alliance.^[32]^ It is suggested that medical institutions at all levels in the medical alliance should build a unified medical information management platform. Patients’ electronic medical records, health records, referral and follow-up information are embedded in it. With the referral of patients, all kinds of information are updated in time to improve information sharing and timeliness. In addition to hardware support, medical staff at all levels should also do a good job of information transfer to avoid repeated consultations, inspections or information omissions.

In this study, these complaints against the medical and nursing illustrated that the current development of the medical alliance had not reached the homogenization. Medical homogenization is an important guarantee to improve the quality of medical service and medical demanders’ experiences. Human resources are the key factor for its realization.^[33]^ It was suggested that the higher-level hospital should make overall arrangements to achieve orderly flow of the staff through various forms. In addition, the internal management mechanism of the medical alliance should also be improved to enhance the enthusiasm of the staff to work in the lower-level hospital. A survey study^[34]^ showed that 55.68% of hospital doctors never had any work-related interaction with general practitioners. The phenomenon was also confirmed in this study. This should draw the attention of administrators in the higher-level hospital.

The Population–Capacity–Process model was derived from a study^[35]^ suggesting that the failure of patient-transition plan could often be attributed to neglect of 1 or more of its 3 essential components (vaguely defined the population(s) to be served, capacity unsuited to patient needs, unreliable process for accessing services).^[36]^ In this study, the caregivers suggested that the large-scale comprehensive tertiary hospitals should focus on treatment, and the lower-level hospitals should focus on rehabilitation and care. Relevant institutions should understand the expectations of medical demanders, clarify the functional orientation of medical institutions at all levels. The purpose of this was to distribute patients to appropriate providers, so that the service content of the medical institution could maximize to meet the current medical needs of patients after the downward referral, solve the unbalanced allocation of medical resources to a certain extent and avoid the failure of the referral. We all know that strong physician-patient alliance is associated with improved outcomes across a range of medical conditions.^[37]^ So, The demands, requirements, and expectations of patients and their families should be protected in the referral processes in any healthcare system.

The more constructive suggestion of this study was to help other developing countries to avoid the problems with healthcare reform similar to those observed in China.

### 4.2. Strengths and limitations

A strength was that it was the first in China to consider the broad range of stroke caregivers’ downward referral experiences related to factors that influence low willingness of the downward referral. There is a philosophical concept: practice determines awareness. So, a further strength was the inclusion of interviewees who had experienced the downward referral in person. Their statements were likely to be more reflective of the reasons for lower downward referral rate than those who had not experienced. However, there were still some limitations. For example, the participants in the study were from the same regional medical alliance, and the results of this study were not fully applicable to others. In addition, the sample size was small, although the 13 participants were recruited purposefully to achieve data saturation.

## 5. Conclusion

Caregivers had enriching experiences about the downward referral. They had more bad experiences due to the imperfect development of medical alliance. Our findings had important policy implications. From the government perspective, our study provided empirical evidence that hospitals at different levels in the medical alliance should establish collaboration standards in terms of system, environment and services to improve medical demanders’ experiences. Moreover, the government should intensify the publicity of medical alliance and referral system. There was also a key point that continuous attention to patients after downward referral couldn’t be less. In general, when establishing the medical-rehabilitation-long-term care chain of universal trust, it was suggested to follow the principle of adjusting measures to local conditions and demand-oriented, which would improve the service capacity and efficiency of lower-level hospitals, and improve the referral experiences of patients and their families.

## Acknowledgments

The researchers are most grateful to the family caregivers who shared their experiences and to the nurses who assisted with the selection of the interviewees.

## Author contributions

**Conceptualization:** Yuan Wang, Lijun Xiang, Xiaomei Zhang.

**Data curation:** Yuan Wang.

**Formal analysis:** Yuan Wang, Lijun Xiang, Jian Chen, Yanli Cui, Xiaomei Zhang.

**Funding acquisition:** Xiaomei Zhang.

**Investigation:** Yuan Wang.

**Methodology:** Yuan Wang, Lijun Xiang, Jian Chen, Yanli Cui, Fengwen Wang, Xiaomei Zhang.

**Project administration:** Yuan Wang, Xiaomei Zhang.

**Resources:** Xiaomei Zhang.

**Supervision:** Fengwen Wang, Xiaomei Zhang.

**Writing – original draft:** Yuan Wang.

**Writing – review & editing:** Yuan Wang, Lijun Xiang, Jian Chen, Yanli Cui, Fengwen Wang, Xiaomei Zhang.
